# Shifting power dynamics in global health governance: a challenge and an opportunity for Asia and the Global South

**DOI:** 10.1136/bmjgh-2025-021565

**Published:** 2025-11-23

**Authors:** Neethi Varadaraja Rao, Priyanka Tomar, Ilona Kickbusch, Helen Clark, K Srinath Reddy, Priya Basu, Indu Bhushan, Jae Kyoun Kim, Sandhya Venkateswaran, Nadine Monteiro

**Affiliations:** 1 Centre for Social and Economic Progress, New Delhi, DL, India; 2 Graduate Institute of International and Development Studies, Geneva, GE, Switzerland; 3 Alliance For Health Policy and System Research, Geneva, GE, Switzerland; 4 The Helen Clark Foundation, Auckland, New Zealand; 5 Public Health Foundation of India, New Delhi, India; 6 The Pandemic Fund, World Bank Group, Washington, District of Columbia, USA; 7 Pahlé India Foundation, Delhi, India; 8 Asian Development Bank, Mandaluyong, NCR, Philippines

**Keywords:** Global Health, Health systems, Pandemic Preparedness, Universal Health Care

**Summary box**

- This commentary examines how recent events, including shifts in development aid and trust in global institutions, create both challenges and opportunities for the Global South to reshape health governance.
- We identify concrete mechanisms through which Asian and Global South countries can strengthen collective health governance, including leveraging non-health multilateral forums, sharing innovations and developing new financing models.
- Drawing from successful examples across the region—from Thailand’s universal health coverage to India’s digital health infrastructure—we demonstrate how South-South cooperation can drive sustainable health system strengthening.
- We propose a vision for health regionalism that begins with strengthening domestic capacity while building towards more equitable global partnerships that transcend traditional North-South dynamics.

##  Background

The growing intersection between geopolitics and health was underscored by the COVID-19 pandemic, which exacerbated the pressures on global health institutions worldwide.^1^ Even as countries retreat into national security approaches, cross-country health challenges such as pandemic preparedness, migration and climate resilience are becoming increasingly salient, underlining the need for global solidarity. The impacts of these polycrises are exacerbating the resource constraints and capacity gaps that have hindered accelerated progress on the Sustainable Development Goals (SDGs) agenda. The ongoing flux in the existing global order has the most acute impacts in countries of the Global South, but it also presents an opening to exert their agency in ways that reshape the architecture of global health governance.

### Regional cooperation on health as a strategic lever

For many countries in Asia and other parts of the Global South, the evolution of the current landscape suggests the need to build robust regional collaborative mechanisms aimed at pooling resources and backstopping variable State health system capacities. Like-minded countries can collaborate more closely within their regions, share capacities and develop localised financing and regulatory structures to sustain progress around SDGs and other global health outcomes. Regional cooperation can thus provide a mechanism for the expression of global solidarity at a time when traditional global health institutions are facing headwinds.

The Global South, particularly emerging economies in Asia, is no longer content to be seen as a recipient of global health solutions. These countries are pushing for a reconfiguration of multilateralism—one that is inclusive, equitable and responsive to their developmental trajectories. In this context, regional collaboration is not just a fallback; it is a strategic lever for influence, sustainability and self-reliance. Additionally, it becomes important to ask the right kind of questions. Instead of asking how Asia can replicate existing regional health governance models, we must ask what kind of regionalism best suits Asia’s needs at this moment. This means designing institutions that are less about formality and more about function, less about harmonisation and more about convergence and less about external validation and more about internal cohesion.

This commentary emerged from the discussions at a session of the Regional World Health Summit held in New Delhi in April 2025, jointly convened by The Asian Collective for Health Systems and the Asian Development Bank. The session was aimed at: (a) deliberating on how countries in the South and Southeast Asia region are responding to global and regional pressures with respect to health and (b) examining policy roadmaps to develop cooperative processes to sustain and strengthen global and regional health institutions. Panellists discussed the institutional and governance arrangements necessary within countries and regionally across countries to address health priorities and how the Asian experience can contribute to contemporary global health discourse and diplomacy.

## Avenues for regional and South-South cooperation

Several pathways for strengthening collective health governance and sustaining global solidarity emerged from the discussions, envisaging greater Global South influence and a dynamic landscape combining national, regional and global systems and interests.

### Prioritising health in non-health-specific multilateral and regional forums

Mainstreaming health in alternative multilateral forums has increasingly become a hallmark of Global South leadership. Historically, institutions such as the World Trade Organization provided crucial platforms where Global South countries united to negotiate important outcomes, like the coalition-driven Trade-Related Aspects of Intellectual Property Rights exemptions that enabled greater access to affordable medicines during health crises.^2 3^ This demonstrated the power of collective action within established global governance frameworks.

Today, this spirit of coalition-building and agenda-setting is being carried forward and expanded into new arenas. The G20, particularly under the leadership of Global South countries like India, Indonesia, Brazil and South Africa, has emerged as a vital forum to bring development issues—including health—into sharper focus on the global stage.^4^,^6^ Asia, with its demographic vitality, economic momentum and optimism, holds particular promise in this evolving landscape.^7^

Regional institutions such as Association of Southeast Asian Nations (ASEAN), Brazil, Russia, India, China and South Africa (BRICS), South Asian Association for Regional Cooperation (SAARC) and Bay of Bengal Initiative for Multi-Sectoral Technical and Economic Cooperation (BIMSTEC) have traditionally given limited attention to health within their broader agendas.^8^ However, there is growing recognition that these platforms hold significant untapped potential to foreground health issues and foster stronger regional collaboration. Meanwhile, newer multilateral development banks like the Asian Development Bank and the African Development Bank have actively prioritised regional health and development agendas, becoming key players in financing health system strengthening and pandemic preparedness in the region.^9 10^ By integrating health more centrally within regional institutions, member countries can enhance cooperation, mobilise resources more effectively and align policies to address shared health challenges. Strengthening the capacity of these regional bodies is essential for managing cross-border health threats and complementing global health governance, offering a more resilient and coordinated approach to public health across regions.

### Sharing innovations and building collective capacity

There are promising examples of innovation and systems reform across the Global South. Asia alone has several examples—from Thailand’s universal health coverage reforms, India’s advancements in digital public infrastructure for health, to Sri Lanka’s longstanding investments in primary healthcare. Regional or plurilateral platforms can play a catalytic role in systematically capturing, contextualising and disseminating these models to other settings. In addition to knowledge exchange, regional and South-South cooperation can strengthen core health system functions. For instance, joint efforts in disease surveillance, health workforce development and regulatory harmonisation can yield efficiencies and bolster resilience.^11 12^

The urgency of building collective capacity was made clear during the COVID-19 pandemic, which exposed the limitations of global mechanisms such as COVAX in ensuring timely and equitable access to vaccines for low- and middle-income countries.^13^ Institutions like the Asian Development Bank have since taken active steps to address these gaps through initiatives like Asia Pacific Vaccine Access Facility. While regions like Latin America and Africa have made significant strides in this direction through Pan American Health Organization (PAHO) and Africa Centres for Disease Control and Prevention (ACDC), respectively, Asia can draw valuable lessons from these models as it lays the groundwork for its own robust regional health architecture.

Such models underscore the added value of regional collaboration, particularly for smaller states where shared services—such as regional disease control centres—can provide cost-effective alternatives to fragmented national investments. Furthermore, regional cooperation opens opportunities for structured co-investment strategies with high-income partners in areas like workforce training and health infrastructure. For instance, given the projected health workforce shortages in many countries of the Global North, structured partnerships with Asia and Africa—regions with younger, more dynamic populations—could support global workforce needs while strengthening domestic public health systems.

### Re-thinking financing models for building health resilience

The COVID-19 pandemic catalysed the re-emergence of innovative financing models for global health and preparedness. In its aftermath, financing efforts have increasingly shifted towards innovative co-investment strategies that unite stakeholders from both the Global North and South around shared priorities such as health workforce development and emergency response capacity. These collaborative approaches move beyond traditional donor-recipient dynamics, embracing a broader coalition that includes emerging economies and private sector actors—thereby diversifying both the sources of funding and the expertise brought to the table.^14^

The Pandemic Fund, established through coordinated G20 efforts during the COVID-19 crisis, illustrates this model by supporting country-led efforts in surveillance, laboratory strengthening and workforce capacity, while promoting regional collaboration and ownership through an inclusive governance framework that balances the voices of contributing and recipient countries alike.^15^ This marks a significant evolution in how pandemic preparedness and health security are financed—through partnership, shared responsibility and regional integration.

### Global South: the power of ‘minds and markets’

With the majority of the world’s population concentrated in the Global South, it represents both a vast health workforce and markets for medical goods and technologies. Moreover, the diversity and scale of populations in these regions can create favourable conditions for testing and scaling innovative technologies and service delivery models that can address complex, rapidly evolving health needs. Emerging economies like India, China, Brazil, Indonesia and South Africa are not only significant consumers of pharmaceuticals, vaccines and diagnostics but are also becoming major contributors to their development and manufacturing. Other countries are also looking to build their own manufacturing capacity to minimise dependencies during crises, which can also be supported through South-South cooperation.

While the Global North continues to face acute shortages in the health workforce, the Global South increasingly supplies trained professionals who sustain health systems around the world.^16^ However, this emigration often leaves behind health workforce shortages in the donor countries of the Global South that are not sufficiently redressed.^17^ This is partly because of the absence of equitable frameworks to govern such human resource transfers. Thus, the Global South must deliberately harness the combined power of ‘minds and markets’ to reshape systems and solutions to be more equitable, resilient and representative.

### Health regionalism begins at home: strengthening domestic capacity first

While opportunities for regional cooperation in the Global South are expanding, these will remain fragile without strong domestic foundations. Strengthening domestic health systems must be the cornerstone of any broader health governance strategy. Without robust national capacity, regional and global initiatives risk being unstable.

Asian health systems are contending with a common set of structural challenges: the unresolved burden of communicable diseases alongside a growing epidemic of non-communicable diseases; rapid demographic transitions marked by ageing populations and significant inequities in access and outcomes within countries. These are further exacerbated by fragmented service delivery across primary, secondary and tertiary care, as well as across public and private providers, and weak regulatory oversight, particularly of the private sector.

Additionally, health financing remains a critical constraint. Many countries allocate insufficient public resources to health, and within that, primary care often receives disproportionately low investment. At the same time, external development assistance has at times distorted national priorities, with funding concentrated in vertical programmes that do not align with current disease burdens and contribute to the fragmentation of systems and misaligned incentive structures.

Countries thus need to prioritise health much more, drawing from public and private sources to adequately resource their health systems. Robust domestic health information and data systems can help countries leverage cooperative and knowledge-sharing mechanisms to rapidly build capacity. These capacities can then be directed towards country and regional priorities, including public health surveillance, digitisation, supply chain stability, workforce standardisation, among others.

## Conclusion

To truly shape the global health and development agenda, the Global South must now move from potential to action—with agility, creativity and a bold collective vision. This means transcending the binaries of North and South to forge a more networked, multipolar world order—where influence is earned through innovation, solidarity and strategic coalition-building. Whether through regional platforms or issue-driven alliances, the Global South has the opportunity to lead on solutions that are inclusive, resilient and future-ready. But seizing this moment also demands sustained investment in domestic systems—from public health infrastructure to local manufacturing and digital governance.

## Data Availability

There are no data in this work.
